# Burnout syndrome and coping strategies among professors in the health area

**DOI:** 10.47626/1679-4435-2024-1175

**Published:** 2024-11-14

**Authors:** Gilvana Maria Vieira Xavier, Claudio José dos Santos Júnior, Mara Cristina Ribeiro, Geraldo Magella Teixeira

**Affiliations:** 1 Centro de Ciências da Saúde, Universidade Estadual de Ciências da Saúde de Alagoas, Maceió, AL, Brazil; 2 Centro de Ciências Integradoras, Universidade Estadual de Ciências da Saúde de Alagoas, Maceió, AL, Brazil; 3 Faculdade de Saúde Pública, Universidade de São Paulo, São Paulo, SP, Brazil

**Keywords:** psychological exhaustion, teachers, psychological adaptation, burnout, psychological, stress, psychological, esgotamento psicológico, docentes, adaptação psicológica, esgotamento profissional, estresse psicológico

## Abstract

**Introduction:**

Burnout syndrome is a condition resulting from chronic exposure to interpersonal stressors in the workplace. Faculty in the health field are particularly susceptible to burnout syndrome due to frequent emotional stress.

**Objectives:**

To identify an association between the dimensions of burnout syndrome and the coping strategies these faculty have adopted.

**Methods:**

An observational, analytical and cross-sectional study was conducted in 2021 with 164 faculty from a public university. The Maslach Burnout Inventory and the Brief-Coping Orientation to Problems Experienced were used. The analysis included descriptive statistics and Spearman correlation, using SPSS 16.0.

**Results:**

Emotional exhaustion was high for 54.88% of the sample. Depersonalization scored high for 97.57% of the participants, whereas personal accomplishment was high for 100%. These results are no indication of burnout syndrome, which requires high scores for emotional exhaustion and depersonalization and low scores for personal accomplishment. Active coping, planning, and positive reframing were the most used strategies, which are aimed at solving problems and are considered protective against this condition. Denial, behavioral disengagement, and substance use, which are focused on avoidance and emotion, with potentially negative effects, were the least used. The correlations between the dimensions of burnout syndrome and the coping factors were weak or very weak.

**Conclusions:**

Although burnout syndrome was not identified, the sample was at a high risk of becoming ill due to emotional exhaustion. The correlations between the dimensions of burnout syndrome and the coping factors were weak.

## Abstract (pt)

**Introdução:**

A síndrome de *burnout* é um adoecimento resultante da exposição crônica a estressores interpessoais no ambiente de trabalho. Docentes da área de saúde estão particularmente suscetíveis a essa síndrome devido às pressões emocionais frequentes.

**Objetivos:**

Identificar a associação entre as dimensões da síndrome de *burnout* e as estratégias de *coping* adotadas por docentes universitários da área de saúde.

**Métodos:**

Realizou-se um estudo observacional, analítico e transversal em 2021, com a participação de 164 docentes de uma universidade pública. Foram utilizados os instrumentos Maslach Burnout Inventory e Brief-Coping Orientation to Problems Experienced. A análise incluiu estatística descritiva e correlação de Spearman, utilizando o SPSS versão 16.0.

**Resultados:**

A exaustão emocional foi elevada para 54,88% da amostra. A despersonalização teve pontuação alta para 97,57% dos participantes, enquanto a realização profissional foi considerada elevada para 100%. Esses resultados não indicam a presença de síndrome de *burnout*, que requer alta pontuação para exaustão emocional e despersonalização e baixa pontuação para realização profissional. As estratégias mais utilizadas foram *coping* ativo, planejamento e reinterpretação positiva, as quais são voltadas para a resolução de problemas e consideradas protetoras contra a doença. As menos utilizadas foram negação, comportamento de desprendimento e uso de substâncias, que são focadas na evitação e emoção, com potencial de efeitos negativos. As correlações entre as dimensões da síndrome de *burnout* e os fatores de *coping* foram fracas ou muito fracas.

**Conclusões:**

Embora a síndrome de *burnout* não tenha sido identificada, a amostra apresentou alto risco de adoecimento devido à exaustão emocional. As correlações entre as dimensões da síndrome de *burnout* e os fatores de *coping* foram fracas.

## INTRODUCTION

Burnout syndrome (BS) is a condition resulting from repeated, long-term exposure to interpersonal stressors in a professional setting.^1-4^ In several international studies, incidence remains uncertain, depending on the population studied. This condition is more common among professionals who work directly with people and are exposed to frequent emotional stress, especially educators, physicians, nurses, and police officers.^1,2,5^

In view of the severe effects of BS on workers, both preventive and treatment strategies can be implemented. The specialized literature states that preventive strategies are necessary^1,2^ and, therefore, should include different stages aimed at preventing and/or delaying the onset of BS. These include organizational adjustments, such as changes in the physical space and management, and coping strategies, which are cognitive and behavioral efforts that employees make to deal with, reduce, or tolerate specific demands that threaten or exceed their personal resources.^6^

This study sought to investigate the relationship between the dimensions of BS and the coping strategies faculty in the health field at a public university in the northeast of Brazil have adopted. The primary objective was to identify the association between the dimensions of BS and the coping strategies these faculty have used.

## METHODS

This is a cross-sectional, analytical, observational study conducted at a public university in Alagoas, a state in northeastern Brazil.

The sample was drawn by non-probabilistic sampling for convenience, and consisted of active faculty who agreed to participate in the study, following electronic recruitment sent to their institutional e-mail address.

We used three data collection instruments: a structured questionnaire to identify the sociodemographic and professional profile of the sample and two validated instruments, the Maslach Burnout Inventory (MBI) to assess BS^7^ and the Brief-Coping Orientation to Problems Experienced (Brief-COPE) scale to assess coping strategies.^8^

The dependent variables of interest were the three dimensions of BS from the MBI: emotional exhaustion (EE), depersonalization (DP) and personal accomplishment (PA). The independent variables were the Brief-COPE coping categories: active coping, planning, use of instrumental support, use of emotional support, religion, positive reframing, self-blame, acceptance, venting, denial, self-distraction, behavioral disengagement, substance use, and humor.

The association between coping strategies and the dimensions of BS was investigated using Spearman’s correlation to find significant correlations (p < 0.05). Spearman’s correlation was used after the Shapiro-Wilk test, revealing that some of the variables of interest did not have a normal distribution.

All statistical analyses were carried out using SPSS.

The Ethics Research Committee approved this study with opinion No. 4.545.726 and Certificate of Submission for Ethical Appraisal 37106920.2.0000.5011.

## RESULTS

Invitations to participate in the study were sent to 292 faculty and 164 (56%) responded. The results showed that women predominated (75.6%; n = 124) in this sample. Most participants (59.8%; n = 98) were married, and brown (45.1%; n = 74) or white (45.1%; n = 74) skin prevailed. Additionally, 73.2% (n = 120) reported having children.

The mean age of the participants was 44.55 years (standard deviation [SD] = 8.68), with a mean length of service of 15.74 years (SD = 8.55) as educators. Workloads of 40 to 60 hours accounted for 42.7% (n = 70) of the sample. The full sociodemographic profile of the sample is shown in Table 1.

**Table 1:** Sociodemographic and professional profile of the sample

Variables			Total (n = 164)	
			n	%
Sex				
Female			124	75.6
Male			40	24.4
Marital status				
Married			98	59.8
Single			34	20.7
Divorced			26	15.9
Widowed			4	2.4
Refused to answer			2	1.2
Ethinicity/skin color				
White			74	45.1
Brown			74	45.1
Black			8	4.9
Indigenous			2	1.2
Yellow			2	1.2
Refused to answer			4	2.4
Children				
Yes			120	73.2
No			44	26.8
Years since graduation				
5 to 9			8	4.9
10 to 14			38	23.2
15 or more			118	72.0
Professional degree				
Nursing			38	23.2
Occupational therapy			22	13.4
Physical therapy			20	12.2
Audiology			10	6.0
Medical Science			6	3.7
Other			68	41.5
Weekly workload (hours)				
20			14	8.5
30			10	6.1
40			50	30.5
40 to 60			70	42.7
> 60			18	11.0
Refused to answer			2	1.2
Monthly income (BMW)				
1 to 4			22	13.4
5 to 6			40	24.4
6 to 10			52	31.7
> 10			42	25.6
Refused to answer			8	4.9
	Low	High	Mean	SD
Age	32	66	44.55	8.686
Length of service	2	46	15.74	8.550
Length of service at this university	1	31	11.45	7.434

% = percentage; n = number of participants; BMW = Brazilian minimum wage; SD = standard deviation.

### HOW THE DIMENSIONS OF BS OCCUR IN FACULTY

**Figure 1:**
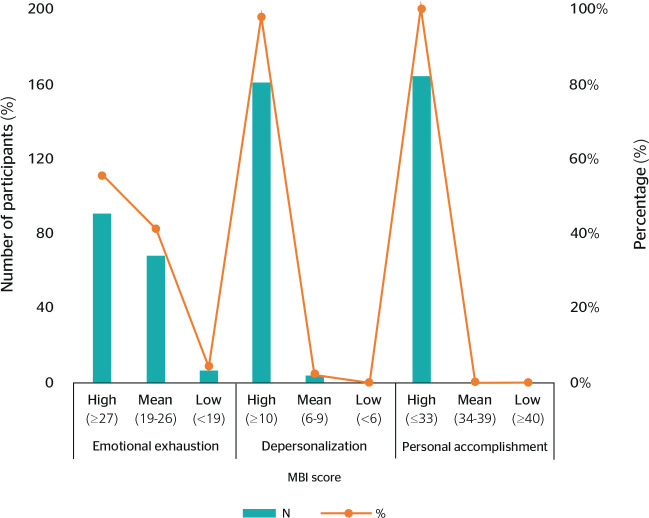
Distribution of the sample according to the Maslach Burnout Inventory (MBI).

The surveyed faculty had a high level (55%) in the EE dimension. The DP dimension also scored highly among 98% of the faculty. The PA dimension showed that 100% of the faculty were personally accomplished. Figure 1 shows the results for each item in the three dimensions of the BS.

### COPING STRATEGIES ADOPTED BY FACULTY

As for coping strategies, the participants in the survey reported that problem-focused strategies were the most frequently used to manage stressors in the workplace. Planning was the factor with the highest mean (M) (M = 4.46 and SD = 1.31), then active coping (M = 4.33 and SD = 1.44), and positive reframing (M = 4.05 and SD = 1.54). On the other hand, strategies focused on emotion and avoidance were the least used, such as religion (M = 3.76 and SD = 1.95), acceptance (M = 3.27 and SD = 1.55), use of instrumental support (M = 3.18 and SD = 1.69), use of emotional support (M = 3.09 and SD = 1.84), self-blame (M = 3.02 and SD = 1.43), venting (M = 2.79 and SD = 1.78), self-distraction (M = 2.70 and SD = 1.57), humor (M = 1.94 and SD = 1.83), denial (M = 1.17 and SD = 1.50), behavioral disengagement (M = 0.71 and SD = 1.15), and substance use such as alcohol and drugs (M = 0.31 and SD = 0.87).

Table 2 shows a descriptive analysis of the Brief-COPE and the mean scores for each dimension.

**Table 2:** Descriptive statistics for the dimensions of coping strategies

Dimensions of coping strategies	M	SD	low-high
Active coping	4.33	1.440	0-6
Planning	4.46	1.312	0-6
Use of instrumental support	3.18	1.695	0-6
Use of emotional support	3.09	1.842	0-6
Religion	3.76	1.948	0-6
Positive reframing	4.05	1.542	0-6
Self-blame	3.02	1.427	1-6
Acceptance	3.27	1.551	0-6
Venting	2.79	1.784	0-6
Denial	1.17	1.501	0-6
Self-distraction	2.70	1.572	0-6
Behavioral disengagement	0.71	1.156	0-4
Substance use	0.31	0.873	0-4
Humor	1.94	1.631	0-6

M = mean; SD = standard deviation.

### CORRELATION BETWEEN THE DIMENSIONS OF BS AND COPING STRATEGIES

In the Spearman correlation analysis between the dimensions of BS and the coping factors, weak or very weak correlations were observed.

Statistically significant coping factors associated with the dimensions of BS were: active coping, planning, use of instrumental support, use of emotional support, positive reframing, self-blame, acceptance, denial, behavioral disengagement, and substance use.

We found a weak positive correlation between self-blame and denial strategies and the EE dimension, while behavioral disengagement had a very weak relationship. No correlation was found between substance use and the EE dimension. The EE dimension was weakly negatively correlated with the active coping strategy, and very weakly correlated with acceptance.

The PD dimension showed a very weak positive correlation with the strategies of instrumental support, substance use, and positive reframing, a weak correlation with denial and no correlation with emotional support. No negative correlation was found between this dimension and the coping variables.

The PA dimension showed a moderate positive association with denial and a very weak association with behavioral disengagement. Conversely, PA correlated moderately with active coping and weakly with planning.

Table 3 shows the results of the correlations found between coping factors and the dimensions of BS.

**Table 3:** Correlation matrix between the dimensions of burnout syndrome and coping factors

Coping strategies	Dimensions of burnout		
	EE	DP	PA
Active coping (n = 164)			
Spearman’s Rho (ρ)	-0.23	0.04	-0.21
p-value	0.0025	0.6357	0.0058
Planning (n = 164)			
Spearman’s Rho (ρ)	-0.14	0.15	-0.16
p-value	0.0721	0.0602	0.0375
Use of instrumental support (n = 164)			
Spearman’s Rho (ρ)	0.01	0.31	0.00
p-value	0.8639	0.0001	0.9681
Use of emotional support (n = 164)			
Spearman’s Rho (ρ)	0.04	0.38	-0.04
p-value	0.5730	0.0000	0.6558
Religion (n = 164)			
Spearman’s Rho (ρ)	0.01	0.13	-0.06
p-value	0.8864	0.1012	0.4353
Positive reframing (n = 164)			
Spearman’s Rho (ρ)	-0.08	0.29	-0.05
p-value	0.2975	0.0001	0.5453
Self-blame (n = 164)			
Spearman’s Rho (ρ)	0.17	-0.01	0.04
p-value	0.0261	0.9040	0.5872
Acceptance (n = 164)			
Spearman’s Rho (ρ)	-0.28	0.07	0.08
p-value	0.0004	0.3977	0.2860
Venting (n = 164)			
Spearman’s Rho (ρ)	0.01	0.13	-0.01
p-value	0.8548	0.1033	0.8607
Denial (n = 164)			
Spearman’s Rho (ρ)	0.26	0.24	0.21
p-value	0.0009	0.0021	0.0067
Self-distraction (n = 164)			
Spearman’s Rho (ρ)	-0.01	0.13	0.13
p-value	0.8739	0.1012	0.0880
Behavioral disengagement (n = 164)			
Spearman’s Rho (ρ)	0.20	0.05	0.26
p-value	0.0091	0.5173	0.0007
Substance use (n = 164)			
Spearman’s Rho (ρ)	0.37	0.19	0.13
p-value	0.0000	0.0168	0.1091
Humor (n = 164)			
Spearman’s Rho (ρ)	0.03	0.12	-0.02
p-value	0.7442	0.1403	0.8306

DP = depersonalization; EE = emotional exhaustion; PA = personal accomplishment; ρ = Spearman’s correlation coefficient.

## DISCUSSION

The response rate for the study was 56% (164 respondents), predominantly women, which can be due to nurses being the largest group of respondents, a category in which women represent more than 85% of these professionals in Brazil.^9^

In addition, most were married (59.8%) and had children (73.2%). Andolhe et al.^10^ point out that the marital status was a protective factor for BS.^10^ Married workers are generally more psychologically mature and have more stable lifestyles, with less symptoms of BS.^11^

The mean age (44.55 years) of the participants shows that this is a sample of adult and experienced workers. Some studies show that individuals up to the age of 30 are more susceptible to BS, and that their lived experiences are relevant in assessing stress and the coping strategies they choose.^10^

This study used the MBI to assess the three dimensions of BS separately, based on the score obtained in each one. The EE, DP, and PA dimensions were high and moderate, but no BS could be identified in the sample. According to the MBI manual, to confirm a diagnosis of BS the professional being assessed should score at a high level for EE and DP and at a low level for PA, simultaneously.^12^

However, it is worth noting that in the process of becoming ill, EE is the first dimension to emerge, and when combined with high scores on the DP dimension, it can be an indicator of BS in the future.^13^ Thus, the percentages for EE and DP are worrisome, indicating the urgent need for intervention to prevent an onset of BS.

This study showed a high EE, which may be linked to greater participation of women in the sample. Some authors state that women are more susceptible to stress than men and are therefore more likely to have a poorer quality of life. On the other hand, women use the venting strategy more often than men, which also justifies their higher level of stress, as found in the self-report data.^12^

Batista et al.^14^ found a link between increased EE and the nature of teaching. Those faculty who had heavy workload reported that their work affected their personal lives, thus becoming a stressor.

The sample assessed showed high DP, which is an attitude of distancing or indifference to those who should receive their services.^1^ Silva & Oliveira^15^ justify the vulnerability of faculty to stress and BS on the grounds that their job involves other individuals and their families, which exposes them daily to conflicts, dilemmas, and requests for help that they cannot always meet. This study found that high workloads is another contributing factor, which are correlated with negative attitude, especially when they involve direct and continuous contact with people.^11^

High PA may be due to a predominant use of problem-focused coping strategies, or to other variables which were not the aim of the study. A second factor to be considered is that this sample is mainly composed of professionals with a mean age of 44, with more than 15 years of service in the field. Workers under the age of 26 showed lower PA than older workers.^16^

In this study, the absence of BS can be attributed to the use of problem-focused strategies as a protective factor. Braun & Carlotto^16^ point out that the greater the use of problem-focused coping strategies, the greater the likelihood of being able to make decisions capable of resolving the stressors faced and achieving their goals, increasing levels of PA.

As for the coping strategies, we found that active coping, planning, and positive reframing were the most commonly used, all aimed at problem-solving. The least used were denial, behavioral disengagement, and substance use, focused on avoidance and emotion. Similar results were also observed in Pocinho & Capelo^17^ in a study with faculty in Portugal, which showed that the primary coping strategies used were problem-focused, then avoidance or escape strategies, and finally emotion-focused strategies.^17^

Active coping and acceptance showed an inverse correlation with EE. This means that the less the individual accepts the problem as true, the more emotionally drained they are likely to be.

On the other hand, denial, behavioral disengagement, substance use, self-blame, religion, and use of emotional support were positively linked to exhaustion. In other words, workers who use these strategies, avoiding the source of stress, experience greater emotional distress.^18^ The use of emotion-focused strategies involves the emergence of demands and feelings of self-blame related to the negative behaviors and attitudes.^19^

The high prevalence of women in the study may have contributed to the use of emotion-focused strategies. Xavier et al.^18^ investigated BS indicators and coping strategies used by faculty, and their primary findings were greater psychological distress and self-blame among women. These educators tended to use emotion-focused coping strategies more often, such as religion and emotional support.^18^ The literature agrees that emotion-focused coping can generate symptoms that can hinder problem-solving, such as self-blame.^2,18^

Denial was positively associated with EE and DP, which reinforces that the process of emotional exhaustion is driven by behavioral disengagement, gradually causing the individual to lose interest in relationships with other people. These findings confirm the findings of Diehl & Marin^20^ and Carlotto,^11^ authors who point out that denial strategies are harmful to workers. This type of strategy contributes to the onset of BS, as it seeks ways to escape from the problem rather than solve it.

The PA dimension was moderately and positively linked to denial and weakly linked to behavioral disengagement. Although the use of emotion-focused strategies is a predictor of illness and lower PA, this study found that these factors were positively associated. Depending on the stressful situation, faculty can use denial or emotion-focused strategies to deal with the problem without interfering with their PA, as observed in this study. In some contexts, denial may be appropriate for well-being at work, adjusting the escape from places and problems, generating fewer worries, as observed in the analysis of faculty in rural settings.^3^ It can therefore be inferred that, among faculty, denial is a way of preventing feelings of dissatisfaction with particular activities.

Conversely, PA was weakly associated with planning and active coping, both problem-focused strategies. These results are contrary to the findings of this study, which indicates that problem-solving strategies increase PA. Planning consists of thinking about how to confront the stressor, planning active coping efforts to initiate an action or making efforts to remove or circumscribe the stressor.^19,21^ Although these strategies are focused on problem-solving, the weak negative association was not able to interfere with PA, which was found to be high in these faculty.

PA was positively associated with denial, use of instrumental support, and substance use, which are emotion-focused factors, and with positive reframing of the problem, which is a problem-focused strategy, the latter result being contrary to expectations. Diehl & Marin^20^ observed that a positive reappraisal of problems reduces the PA, and was inversely related. However, the correlation presented in this study was very weak.^20^ The use of positive reframing demonstrates an emotional approach to dealing with stressors in the workplace, aiming at maintaining affective balance.

On the other hand, substance use consists of avoiding the problem through the use of chemical substances or alcohol, interfering with the ability of the individual to evaluate situations.^22^ Thus, rather than dealing with the problem, the individual seeks alternatives to escape from the stressful situation. These strategies tend to increase attitudes of indifference as a way of relieving work-related stress. Behavioral disengagement is associated with critical and derogatory attitudes towards their work and the students they assist.^19^

It is important to mention that this study has limitations, including the so-called “healthy worker effect,” which may have influenced the incidence rate of BS. In addition, a sample loss of 46% may indicate that those professionals suffering from BS did not respond to the survey, which may have biased the results.

## CONCLUSIONS

The level of EE, DP and PA was high and moderate, but BS could not be found in the sample. However, high EE, combined with high DP, may be an indicator of BS in the future, signaling the urgent need for intervention to prevent the onset of BS.

The use of problem-focused coping strategies, which prevailed in the study, provides a plausible explanation for why the sample is not suffering from BS, contrary to what is expected in this group of professionals, who are considered vulnerable to the onset of BS.

Another important result, contrary to previous findings, was the association between PA and active coping and planning strategies, both problem-focused, which were inversely but weakly associated. Equally weakly, but positively, PA was associated with emotion-focused strategies, such as denial and behavioral disengagement.
